# Evaluating the accuracy of the Veterans Health Administration’s REACH VET suicide prediction model for legal involved veterans

**DOI:** 10.1038/s44184-025-00167-1

**Published:** 2025-10-18

**Authors:** Alex H. S. Harris, Andrea K. Finlay, Esther L. Meerwijk

**Affiliations:** 1 HSR Center for Innovation to Implementation, Veterans Affairs Palo Alto Health Care System, Palo Alto, CA USA; 2 Stanford–Surgery Policy Improvement Research and Education Center (S-SPIRE), Department of Surgery, Stanford University School of Medicine, Palo Alto, CA USA; 3 National Center on Homelessness Among Veterans, Veterans Affairs Palo Alto Health Care System, Palo Alto, CA USA; 4 Division of Health Systems Science, Department of Medicine, UMass Chan Medical School, Worcester, MA USA

**Keywords:** Disease prevention, Health services, Predictive medicine

## Abstract

The Veterans Health Administration’s (VA) Recovery Engagement and Coordination for Health–Veterans Enhanced Treatment (REACH VET) suicide prevention program uses a model that estimates every patient’s suicide risk. We evaluated predictive accuracy of that model for death by suicide, and separately for the combined outcome of suicide attempt or death, for all VA patients and for patients with criminal legal system involvement who have one of the highest suicide rates among VA users. We found that the model has low accuracy for the prediction of death by suicide (positive predictive value = 0.05% overall, 0.10% for legal-involved; false negative rate = 98.1% overall, 82.4% for legal-involved). For the combined outcome, model accuracy was better and more so for legal-involved veterans, but overall still low. While the REACH VET program has been shown to have benefits, the underlying model that identifies patients at high risk of suicide needs improvement.

## Introduction

Among adults living in the United States (US) in 2021, 7.3% (18.8 million) were military veterans of whom 31.2% (5.9 million) received services from the Veterans Health Administration (VA)^1^. Of the 46,412 US adults who died by suicide in 2021, 14% (6392) were veterans, 38% (2429) of whom were VA users^1^. The suicide death rate for non-veteran US adults is 18 per 100,000 life-years, compared to 30 per 100,000 life-years for all veterans, 39 per 100,000 life years for users of the VA, and much higher for subgroups of VA users^1^. The reasons veterans in general and VA users in particular have elevated rates of suicide have been hypothesized to include factors related to joining the military; experiences before, during, and after military service, such as combat or military sexual trauma; higher burden of physical health challenges including traumatic brain injury; mental health challenges including post-traumatic stress disorder; access to firearms, and other sociodemographic factors^2–5^.

Roughly 0.8% (~46,000) of VA users in Fiscal Year 2023 had recent involvement with the criminal legal system and were served by VA’s Veteran Justice Programs. The Veteran Justice Programs aim to link veterans from criminal legal settings to appropriate healthcare, housing, and other indicated programs^6^. Comprised of two branches, the Healthcare for Re-entry Veterans program primarily serves incarcerated veterans exiting prison and re-entering community living. The Veterans Justice Outreach program primarily serves Veterans who have crisis encounters with law enforcement in the community, have been arrested and are in jail custody, or are being adjudicated or monitored in court settings. Veterans who receive services from these programs, referred to here as “legal-involved veterans”, have among the highest suicide rates among VA patients—over 150 per 100,000 life-years, which is approximately 75 deaths per year^1^. Furthermore, research has shown that legal-involved veterans may have suicide risk factors that differ from other veterans or the general population^7,8^.

In response to veteran suicide rates, VA has made suicide prevention a national priority with several significant programmatic investments directly focused on suicide prevention. These programs include crisis intervention services (e.g., National Suicide Hotline^9^), targeted communication and outreach (e.g., “Don’t wait. Reach out.” PSA campaign), staff training (e.g., VA SAVE), enhanced identification and outreach to high-risk patients (e.g., Recovery Engagement and Coordination for Health–Veterans Enhanced Treatment [REACH VET]^10^), and investments in community prevention (e.g., Staff Sergeant Fox Suicide Prevention Grant Program^11^). VA has also invested in services and programs to address known suicide risk factors and high-risk populations including programs focused on homelessness, criminal legal system involvement, unemployment and financial stress, and firearm safety.

One of VA’s major suicide prevention efforts is the REACH VET program. REACH VET is a nationwide suicide prevention program based on a statistical model that is run monthly to produce estimates of every VA patient’s risk of death by suicide in the next 30 days^12,13^. Patients at the highest risk of suicide (~6000 patients per month) are identified with this statistical prediction model. The REACH VET model is a 61-variable logistic regression model and uses model coefficients that were determined in 2017^10,13^. Suicide Prevention Coordinators at each VA facility communicate with clinicians who reevaluate treatment plans for these patients, conduct outreach, and initiate care enhancements^10^. A quasi-experimental evaluation of the REACH VET program found its implementation to be associated with increases in completed outpatient appointments and proportion of individuals with new safety plans, as well as reductions in mental health admissions, emergency department visits, and suicide attempts. However, the program was not found to be effective in reducing death by suicide or all-cause mortality^10^.

Although the REACH VET program is multifaceted, its overall effectiveness to prevent death by suicide, and perhaps other outcomes, depends to a large degree on how well the underlying statistical model predicts the risk of suicide. The original model was deployed in 2017 and had 381 inputs extracted from the electronic medical record, including sociodemographic variables (e.g., age, marital status, race/ethnicity, homelessness), prior suicide attempts, mental health diagnoses (e.g., depression, substance use disorder), medical diagnoses including pain diagnoses, and health care utilization (e.g., emergency department use, psychiatric discharge), and medication-related variables. The primary method used to assess model accuracy was comparing the suicide rate at different risk tiers to the overall population, which the authors referred to as ‘risk concentration’. As the suicide rate was 30 times higher (1079 per 100,000 life years) in the top 0.1% risk group compared to the overall sample (36 per 100,000 life years) this group of patients was labeled as high risk and became the clinical focus of the REACH VET program^12^. The model was later refined to include only 61 inputs with similar risk concentration. While the risk concentration in the high-risk group was considerably higher, the sensitivity of the model was only 2.2%, meaning 97.8% of the suicides occurred in the ‘lower-risk’ group^13^. It should be noted that other standard metrics of prediction model accuracy, such as the positive predictive value, and accuracy for specific high-risk subgroups were not reported.

The REACH VET model does not include indicators of legal system involvement as inputs, which may be surprising given the elevated risk of suicide attempts and mortality among legal-involved veterans^8^. It is currently unknown if the REACH VET model is more or less accurate for the prediction of death by suicide for legal-involved veterans. Therefore, the primary goals of this study were (1) To determine the performance of the REACH VET risk prediction model for all VA patients in terms of standard accuracy metrics; and (2) To evaluate if the suicide death prediction model is more or less accurate for VA patients with legal involvement. While the REACH VET model was not trained to predict suicide attempts, the REACH VET *program* certainly intends to prevent suicide attempts, as well as suicide deaths^10^. A third goal of this study therefore was to evaluate these two questions for the combined outcome of suicide attempt or death. The results of this study provided a baseline and reference for a second study that evaluated adding predictors associated with legal-involved veterans to the REACH VET model.

## Methods

In this cohort study, we used the current REACH VET methodology to compute suicide risk scores for all VHA patients alive at the start of each of five purposely selected months to capture variation across 2018: March, May, July, September, and November. The SQL scripts we used were originally created to compute historical REACH VET scores and were provided and validated by the VA Serious Mental Illness Treatment Resource and Evaluation Center. Our study was approved by the Stanford University Institutional Review Board and the Research and Development Committee of the VA Palo Alto Health Care System.

### Data definitions

Predictor variables for the REACH VET model are available electronically from VHA’s Corporate Data Warehouse (CDW), which houses all clinical and administrative records for VA patients. To determine whether patients died by suicide we used cause of death data from the VA Mortality Data Repository^14^. To accommodate our third goal, we also determined whether patients attempted suicide. Suicide attempt was based on ICD-10 diagnosis codes and the consolidated records from suicidal behavior and overdose reports and data from the Suicide Prevention Action Network that were available in CDW^15^. Death by suicide and suicide attempts were determined during the 30 days following the index date (e.g., March 1 to March 30). Patients were coded as involved in the legal system if their records in CDW indicated at least one outpatient visit with the Veterans Justice Programs stop codes 591 (Health Care for Re-entry Veterans) or 592 (Veterans Justice Outreach) during the year before the index date.

### Data analyses

The existing model coefficients were applied to each patient’s data to produce a probability that the patient would die by suicide in the next 30 days. The REACH VET program classifies all patients with a REACH VET risk score in the top 0.1% as high risk for death by suicide. Accordingly, we ranked patients by their risk score (probability that the patient would die by suicide) and coded the top 0.1% as the high-risk group. All other patients were coded as lower-risk. We intentionally use the label ‘lower risk’ instead of ‘low risk’, as risk levels among patients outside the high-risk group can still be very high, just not high enough to make it into the top 0.1%. We then calculated the monthly and annual incident rates (outcome per 100,000), the incident rate ratio (IRR) between high- and lower-risk groups, the positive predictive value (PPV; the proportion of patients in the high-risk group who died by suicide) - and the false negative rate (FNR; the proportion of patients who died by suicide who were classified as lower-risk). Ideally, PPV is as close to 1 (one) as possible, but in this context the upper bound is the number of monthly suicides (~180) divided by the size of the high-risk group (~6300), or about 0.028. Ideally, FNR will be as close to 0 (zero) as possible.

We calculated each of these metrics for each month independently. Summary values for incident rate, IRR, PPV and FNR across all five months were obtained through random-effects meta-analysis with the metafor package for R 4.4.0^16^. Simple counts (e.g., total sample size, number of monthly suicides) were summarized with means and standard deviations over the five months. The same analyses were conducted for the combined outcome of suicide attempt or death using the same model coefficients and high-risk group as were used to predict death by suicide. That is, we did not re-train the REACH VET model on the combined outcome of suicide attempt or death. Basic descriptive analyses were done in Python 3.8^17^. Due to the terms of our data use agreements, we cannot report specific cell sizes less than 10. For the many results that included cells <10, we report means and standard deviations over the 5 months.

## Results

### Death by suicide among All VA patients

As presented in Table 1, the mean number of patients included in the REACH VET sample was 6,277,109 (standard deviation of 13,430) per month, the monthly average number of suicides per 100,000 was 2.85 (95% CI 2.66 to 3.06; Supplemental Fig. S1), 34.2 annual deaths per 100,000 or an average of 179 (15.7) per month. There were on average 6276 (15.9) patients in the REACH VET 0.1% high-risk group each month, with an average of 3.4 (1.5) deaths by suicide per month.

**Table 1:** Rates of Death by Suicide and Combined Suicide Attempts or Deaths for All VA Patients and Legal-Involved Patients

	All VA patients			Legal involved patients		
Deaths by Suicide						
	Mean (SD) N per month	Outcomes per 100,000	Mean outcomes (SD) per month	Mean (SD) N per month	Outcomes per 100,000	Mean outcomes (SD) per month
All patients	6,277,109 (13,430)	2.8	179 (15.7)	49,101 (308.7)	6.9	3.4 (1.5)
0.1% high-risk group	6276 (15.9)	54.0	3.4 (1.5)	739 (12.1)	79.0	0.6 (0.5)
Suicide Attempts or Deaths						
	Mean (SD) N per month	Outcomes per 100,000	Mean outcomes (SD) per month	Mean (SD) N per month	Outcomes per 100,000	Mean outcomes (SD) per month
All patients	6,277,109 (13,430)	35.6	2236 (123.3)	49,101 (308.7)	362.2	177.8 (19.3)
0.1% high-risk group	6276 (15.9)	5,175.2	324.8 (29.4)	739 (12.1)	6630.6	50.0 (3.5)

Source/Notes: SOURCE Authors’ analysis of data from the VA Corporate Data Warehouse. NOTES Numbers and rates were averaged over five purposely selected months in 2018.

Presented in Table 2 and Supplemental Fig. S2, the incidence rate was 19.3 (95% CI: 12.0 to 31.3) times higher in the high-risk group compared to the rest of the population. The PPV was 0.00054 (95% CI: 0.00034 to 0.00087), indicating that very few (0.054%) of the patients in the high-risk group died by suicide. The FNR was 0.981 (95% CI: 0.970 to 0.988), meaning that 98.1% of the deaths by suicide were in the lower-risk group. Supplemental Figs. S3–S4 visually display the average of these monthly accuracy metrics.

**Table 2:** Incidence rate ratio, positive predictive value, and false negative rate for deaths by suicide and combined suicide attempts or deaths for all VA patients and legal-involved patients

	All VA patients		Legal-involved patients	
Deaths by Suicide				
	Metric	95% confidence interval	Metric	95% confidence interval
IRR	19.3	12.0 to 31.3	14.0	4.0 to 49.0
PPV	0.00054	0.00034 to 0.00087	0.001	0.0005 to 0.003
FNR	0.981	0.970 to 0.988	0.824	0.573 to 0.972
Suicide Attempts or Deaths				
IRR	169.7	161.0 to 178.9	25.6	22.1 to 29.6
PPV	0.052	0.048 to 0.055	0.068	0.060 to 0.076
FNR	0.855	0.848 to 0.861	0.719	0.688 to 0.747

Source/Notes: SOURCE Authors’ analysis of data from the VA Corporate Data Warehouse. NOTES Accuracy metrics were averaged over five purposely selected months in 2018.

### Death by suicide among legal-involved patients

Summarized in Table 1, an average of 49,101 (308.7) legal-involved veterans were included in the REACH VET sample each month. As presented in Table 1 and Supplemental Fig. S5, the monthly average number of suicides per 100,000 was 6.9 (95% CI 4.30 to 11.14), 83.1 annual deaths per 100,000, or an average of 3.4 (1.5) deaths by suicide per month.

An average of 739 (12.1) legal-involved veterans were identified as high-risk. Legal involved veterans were 0.8% of VA patients but 11.8% of the high-risk group. Of legal involved veterans in the high-risk group, an average of 0.6 (0.5) died by suicide per month. Presented in Table 2, the incidence rate was 14.0 (95% CI: 4.0 to 49.0) times higher in the high-risk group compared to the rest of the population. The PPV was 0.001 (95% CI: 0.0001 to 0.003), indicating that very few (0.1%) of the legal-involved patients in the high-risk group died by suicide. The FNR was 0.824 (95% CI: 0.573 to 0.972), meaning that 82.4% of the deaths by suicide among legal-involved patients were in the lower-risk group. Supplemental Figs. S6–S8 visually display the average of these monthly accuracy metrics.

### Suicide attempt or death among all VA patients

As presented in Table 1 and Supplemental Fig. S9, the monthly average number of suicides or attempts per 100,000 was 35.6 (95% CI 35.0 to 36.3), annual average of 427.2 per 100,000, or on average 2236 (123.3) per month. Presented in Table 2 and Supplemental Fig. S10, the incidence rate of suicide attempts or death was 169.7 (95% CI: 161.0 to 178.9) times higher in the high-risk group compared to the rest of the population.

Among the patients in the top 0.1% of risk, an average of 324.8 (29.4) per month attempted or died by suicide, with a PPV of 0.052 (95% CI: 0.048 to 0.055) and FNR of 0.855 (95% CI: 0.848 to 0.861). This means that 5.2% of the high-risk group died by suicide or attempted suicide and 85.5% of the attempts and deaths by suicide were in the lower-risk group. Supplemental Figs. S11 and S12 visually display the average of the monthly PPV and FNR.

### Suicide attempt or death among legal-involved patients

Summarized in Table 1, among legal-involved patients, an average of 177.8 (19.3) attempted or died by suicide per month, a monthly rate of 362.2 per 100,000, or an annual rate of 4347 per 100,000. Among legal-involved veterans in the high-risk group, there was a monthly average of 6630.6 outcomes per 100,000, or an average of 50.0 (3.5) outcomes per month. Although legal-involved patients were only 11.8% of the high-risk group, they accounted for 17.4% of the true positives (~50).

Presented in Table 2 and Supplemental Fig. S13, the incidence rate of suicide attempts or deaths was 25.6 (95% CI: 22.1 to 29.6) times higher among legal-involved veterans in the high-risk group compared to the rest of the legal-involved veteran population. The PPV was 0.068 (95% CI: 0.060 to 0.076), and the FNR was 0.719 (95%CI: 0.688 to 0.747), visually presented in Supplemental Figs. S14 and S15.

## Discussion

Suicide is notoriously hard to predict and existing risk prediction models are known to have poor predictive accuracy^18^. In this study, we found that the suicide risk prediction model at the core of VA’s REACH VET program produces a more concentrated risk pool compared to the general population of VA patients, however, it still has low PPV and a high FNR for predicting death by suicide. This means that very few people in the high-risk group died by suicide and most of the people who died by suicide were in the lower-risk group. The low accuracy of the model may partially explain that while the only evaluation of the REACH VET program did find positive effects for some outcomes (e.g., increases in completed outpatient appointments, proportion of individuals with new safety plans), it did not find a positive impact on reducing death by suicide^19^. Clearly, the REACH VET program’s effectiveness to prevent death by suicide might be enhanced if the accuracy of the prediction model can be improved – an initiative already under way by VA’s Office of Suicide Prevention^19^.

While legal-involved patients were overrepresented in the high-risk group as identified by the REACH VET model, this did not translate to meaningfully higher accuracy among this patient group. The average number of suicide deaths per month for legal involved veterans was 3.4 overall and 0.6 in the high-risk group. This means that most of the ~150 VA facilities had zero suicide deaths among legal-involved veterans each month or even for the year.

When the outcome was expanded to include suicide attempts or deaths, the accuracy of the model was better in terms of IRR, PPV, and FNR, but still very modest. The model was somewhat more accurate for legal-involved VHA patients compared to other VA patients in terms of predicting death by suicide and attempts. But still, most of the events occurred in the lower-risk group. It is imperative to improve the accuracy of the prediction model to maximize the value of the resources being devoted to intervening with the high-risk group.

Some caveats are important to note. First, these analyses were intended to assess the accuracy of the risk model, not the overall effectiveness of the REACH VET program. Even if the model has low accuracy for predicting death by suicide, that does not mean that the REACH VET program is ineffective or that suicidal patients did not receive guideline-recommended care. In fact, the program has been shown to have benefits other than preventing death from suicide^10^. Second, these analyses used data generated during a time when the REACH VET program was active, meaning that the intervention may have prevented some suicides and attempts in the high-risk group. However, based on the results of an evaluation of the effect of the REACH VET program, which found a 5% reduction in the probability of documentation of a suicide attempt but no effect on deaths by suicide^10^, we believe this effect to be minimal. Third, we used methods that were developed to retrospectively compute historical REACH VET risk scores, as the actual scores as computed by the model at the time (i.e., 2018) were not available. While the methods were validated, their results when applied to data from a different year may deviate somewhat from the actual scores. Fourth, we did not re-train the REACH VET model for combined suicide attempts and death. Given the higher occurrence of suicide attempts compared to deaths by suicide, it is conceivable that higher overall model accuracy can be obtained by re-training the model. Fifth, the VA Office of Suicide Prevention is actively updating the REACH VET model to improve its accuracy^19^. However, until that update is operationally implemented, our results provide an evaluation of the current model, as well as a numeric and methodological baseline for comparison once the new model is available.

Several strategies might help improve the accuracy of the REACH VET risk model overall and/or for high-risk subgroups such as legal-involved veterans. Other modeling strategies^13,20^, enriching the model with contextual variables (e.g., area social determinants of health) or inputs using NLP-extracted variables from clinical progress notes^21,22^, reducing reliance on ICD10 codes as predictors when possible, focusing on prediction within high-risk groups, and changing the time-frame for observing outcomes should all be considered as possible means to improve model accuracy. It might also be helpful to train the model on the more frequent combined outcome of suicide deaths and attempts, or possibly the occurrence of any potentially preventable events, such as inpatient psychiatric admissions. We are currently examining if adding indicators associated with criminal legal involvement will improve the accuracy of the model for this high-risk group. We are also developing and testing a model specifically for legal-involved veterans. Improving prediction model accuracy as well as the effectiveness of downstream program components will help ensure that the tremendous effort involved to operate REACH VET yields maximum benefits for VA patients.

## Supplementary information

Supplementary information

## Data Availability

The United States Department of Veterans Affairs (VA) places legal restrictions on access to veterans’ health care data, which includes both identifying data and sensitive patient information. The analytic data sets used for this study are not permitted to leave the VA network without a Data Use Agreement. For more information, please visit https://www.virec.research.va.gov or contact the VA Information Resource Center (VIReC) at virec@va.gov.
