# Supplementary material for: Evaluating the accuracy of the Veterans Health Administration’s REACH VET suicide prediction model for legal involved veterans
Source: Npj Ment Health Res. 2025 Oct 18;4:53. doi: 10.1038/s44184-025-00167-1 (PMC12535588; doi:10.1038/s44184-025-00167-1)
Supplement: Supplementary file 1 — Supplementary information [file 44184_2025_167_MOESM1_ESM.pdf]

Supplemental Figure S1: Meta-analytic average number of monthly suicide deaths per 100,000.

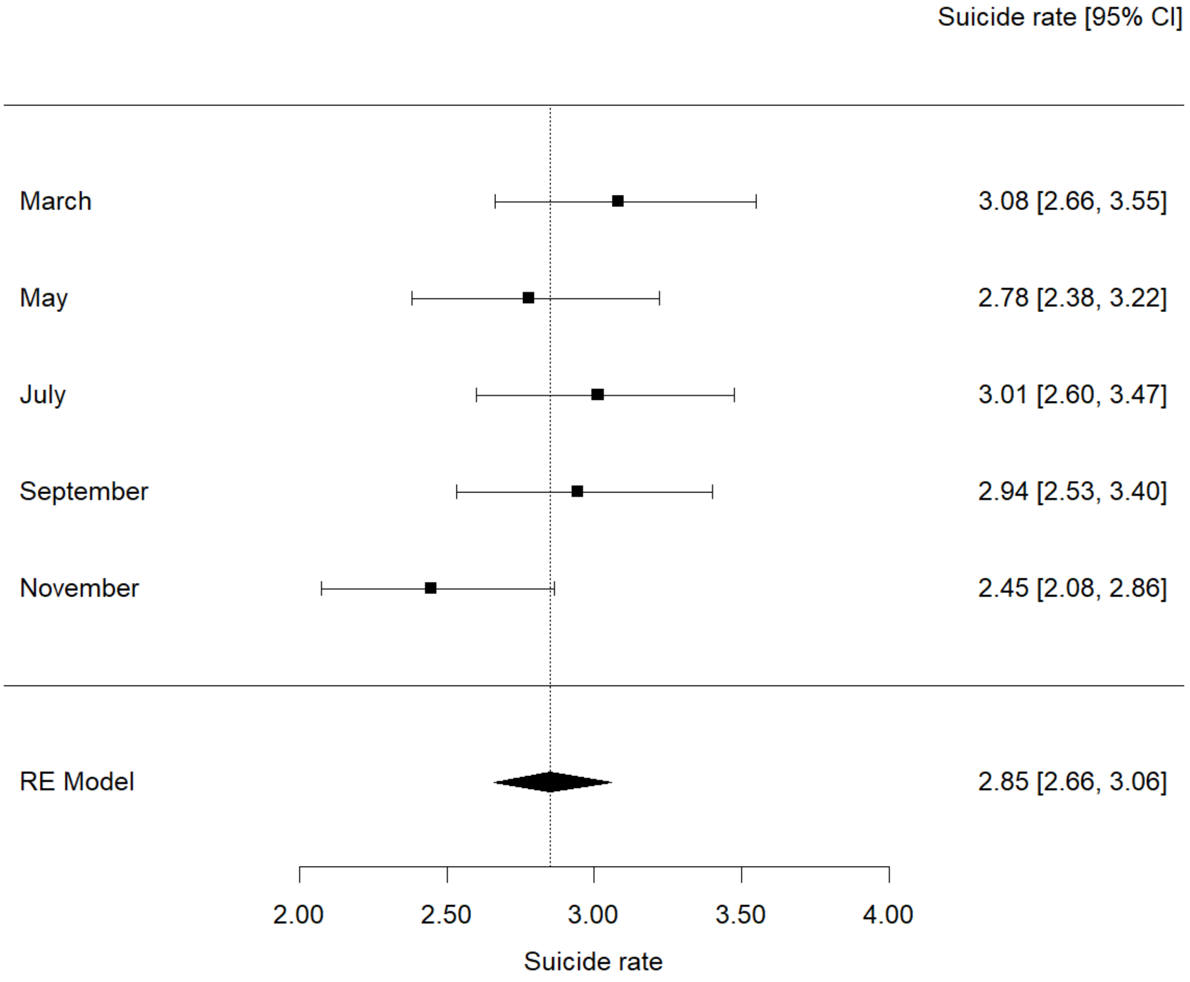

Source/Notes: SOURCE Authors' analysis of data from the VA Corporate Data Warehouse. NOTES The monthly average (2.85) can be multiplied by 12 to get the more common metric of 34.2 annual deaths per 100,000.

Supplemental Figure S2: Meta-analytic monthly average incident rate ratio ("risk concentration") of high-risk group (top 0.1% of risk) to the rest of the sample.

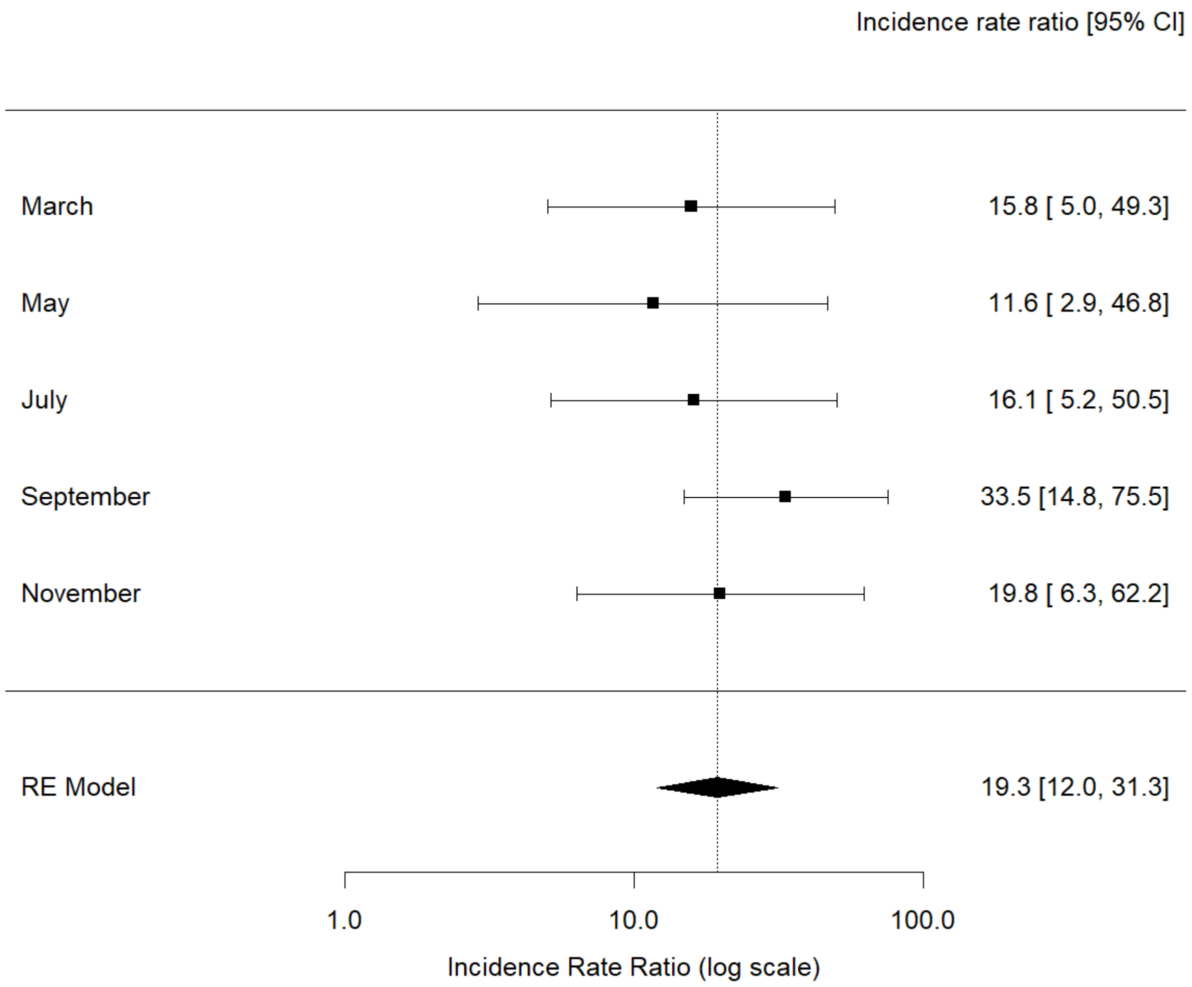

Source/Notes: SOURCE Authors' analysis of data from the VA Corporate Data Warehouse. NOTES In this context, the incident rate ratio signifies that the high-risk group died by suicide 19.3 time more frequently than the rest of the sample.

Supplemental Figure S3: Meta-analytic average positive predictive value (PPV) of being in the high-risk group (top 0.1% of risk).

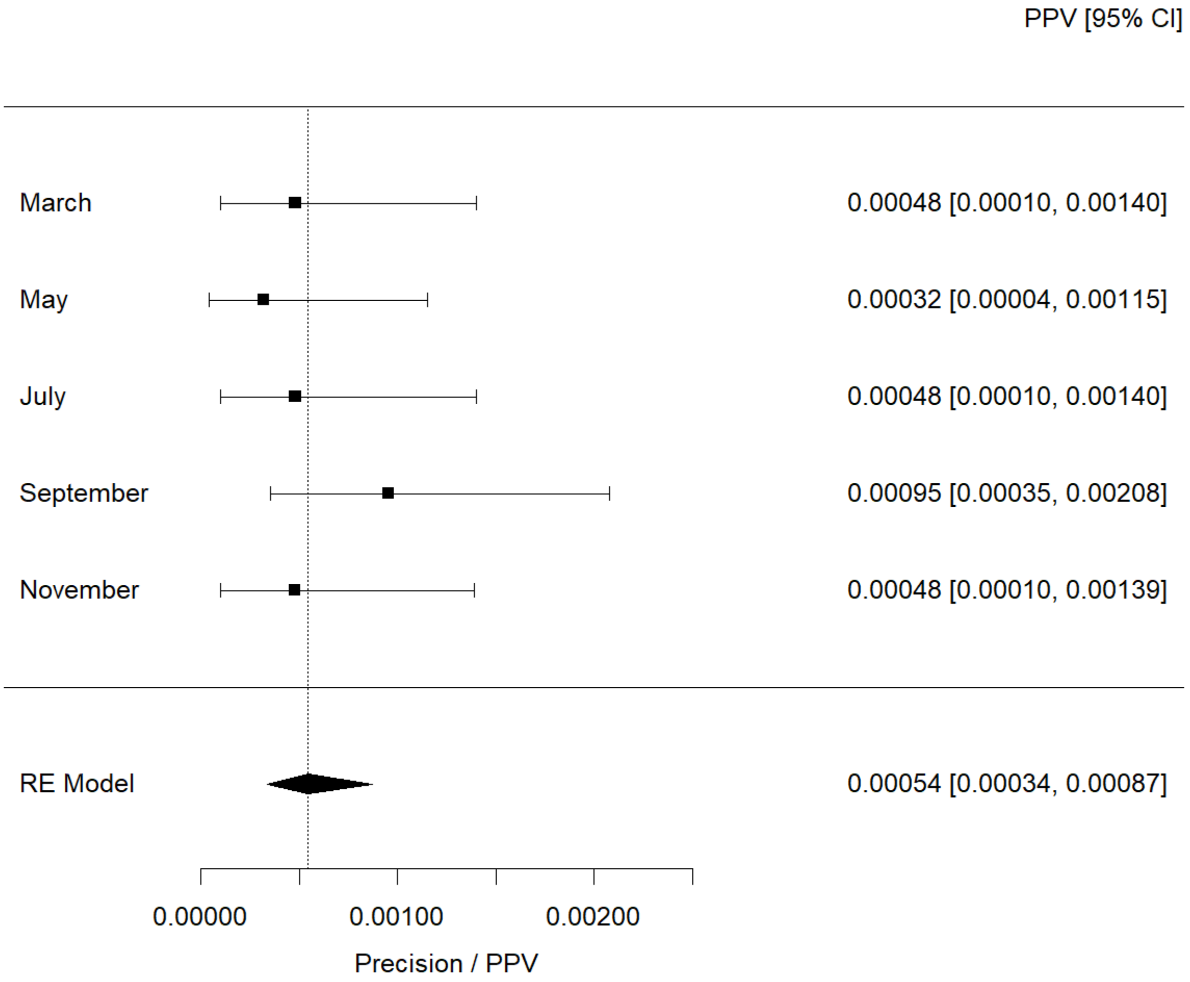

Source/Notes: SOURCE Authors' analysis of data from the VA Corporate Data Warehouse. NOTES In this context, the positive predictive value signifies that 0.054% (less than 10) of those in the high-risk group died by suicide.

Supplemental Figure S4: Meta-analytic average of the false negative rate (FNR).

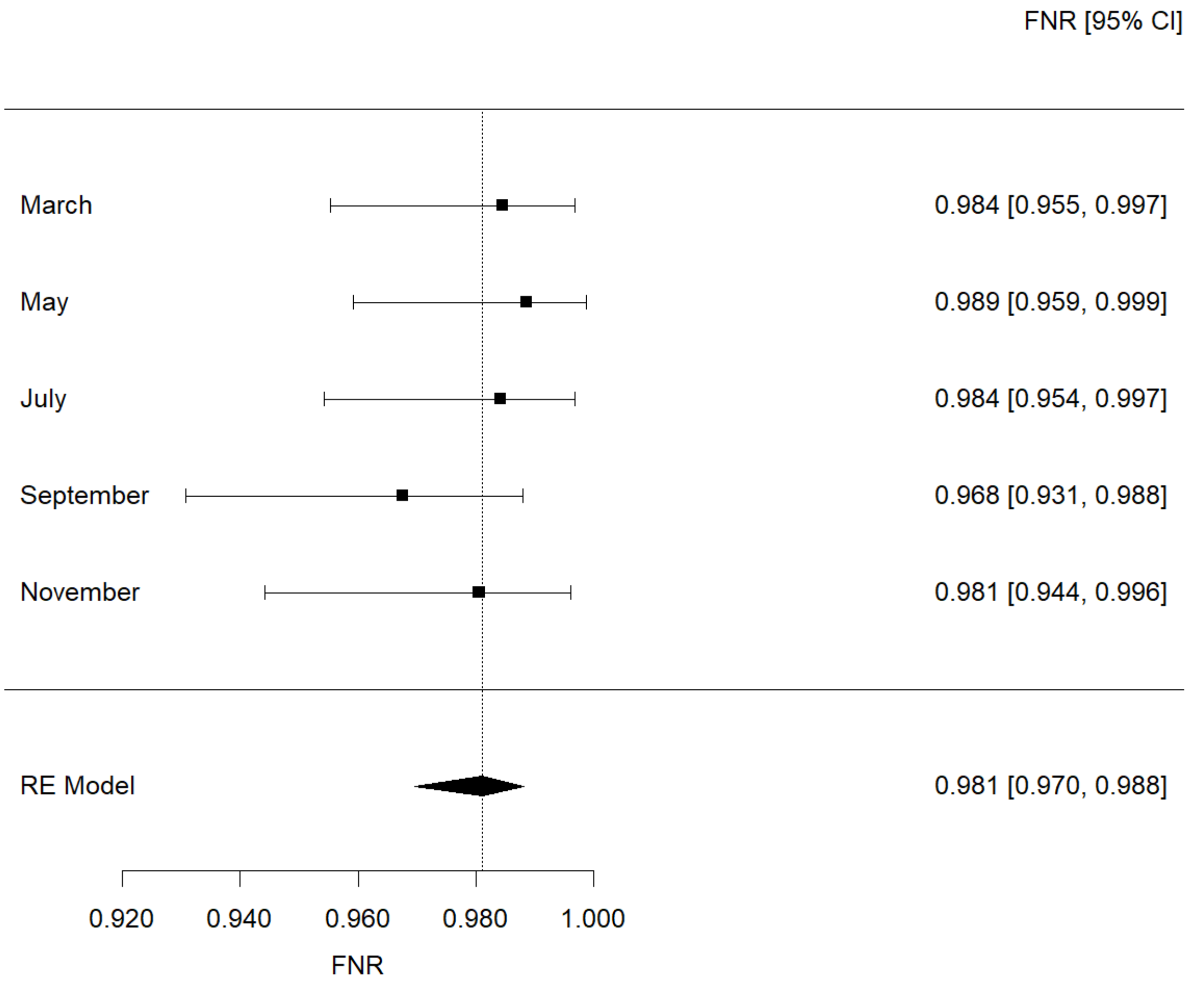

Source/Notes: SOURCE Authors' analysis of data from the VA Corporate Data Warehouse. NOTES In this context, the FNR signifies that 98.1% of the deaths by suicide were in the lower-risk group.

Supplemental Figure S5: Meta-analytic average number of monthly suicide deaths per 100,000 for legal-involved veterans.

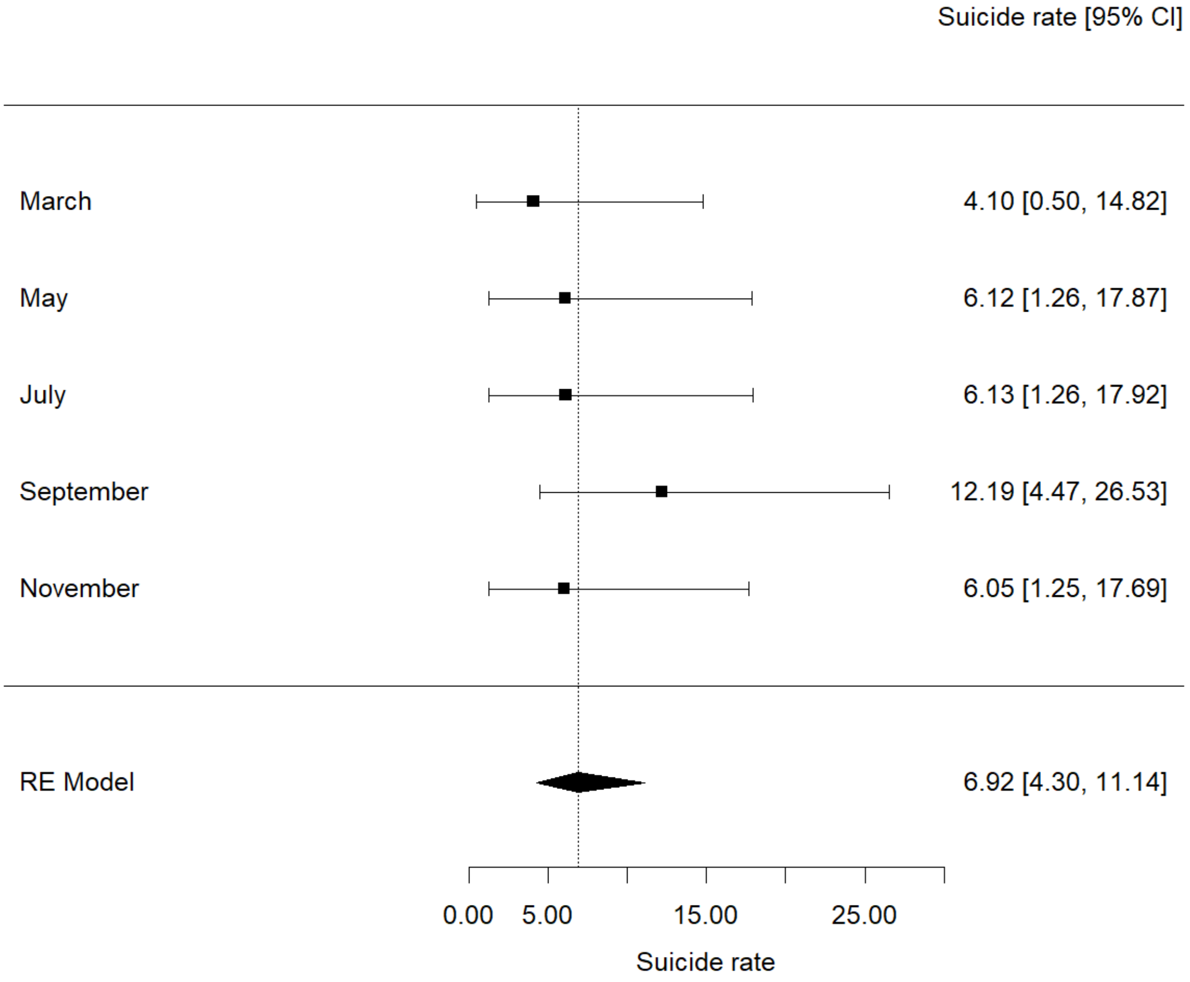

Source/Notes: SOURCE Authors' analysis of data from the VA Corporate Data Warehouse. NOTES The monthly average (6.92) can be multiplied by 12 to get the more common metric of 83.1 annual deaths per 100,000.

Supplemental Figure S6: Meta-analytic monthly average incident rate ratio ("risk concentration") of high-risk group (top 0.1% of risk) to the rest of the sample of legal-involved patients.

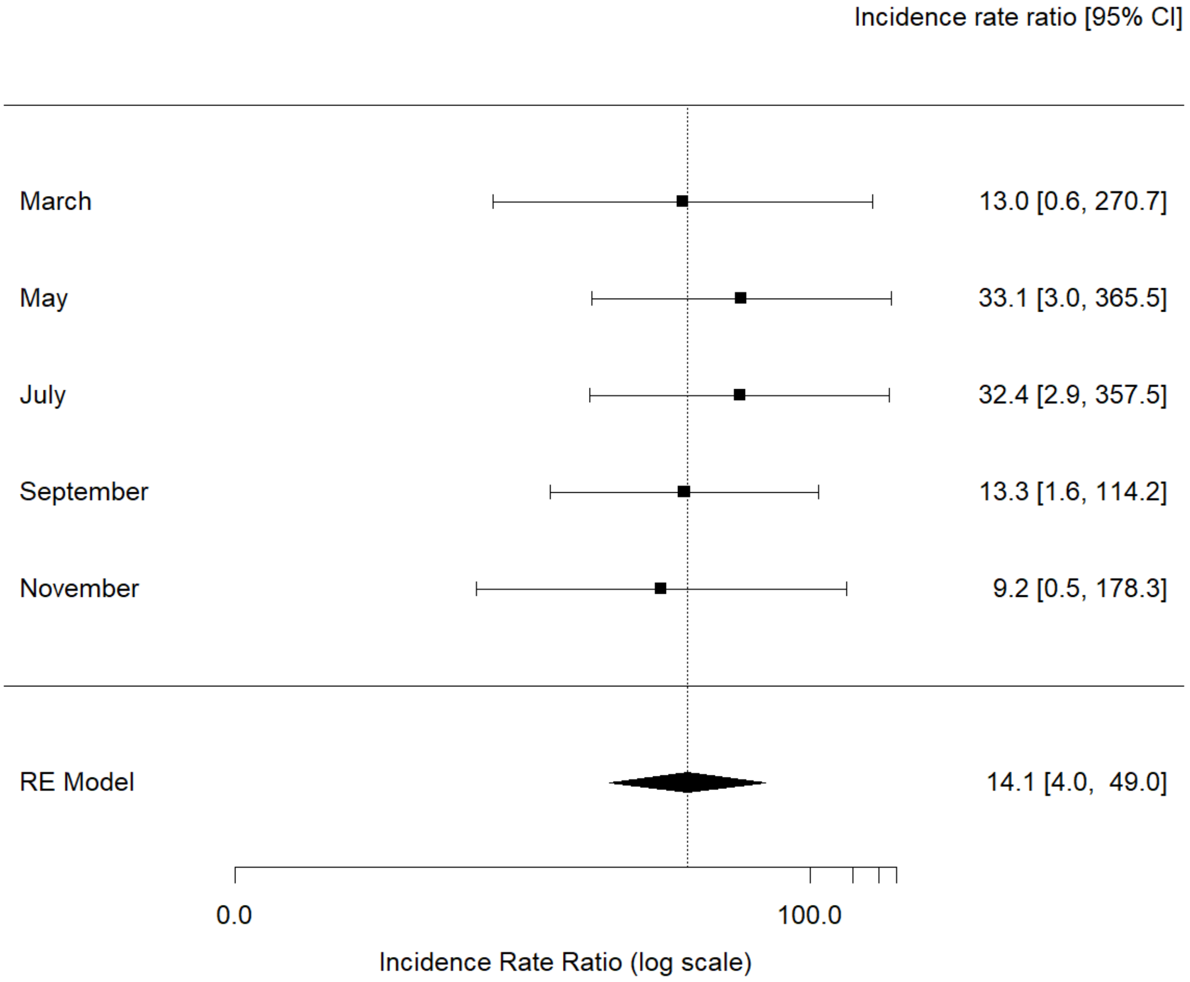

Source/Notes: SOURCE Authors' analysis of data from the VA Corporate Data Warehouse. NOTES In this context, the incident rate ratio signifies that the high-risk group of legal-involved patients died by suicide 14.1 time more frequently than the rest of the sample of legal-involved patients.

Supplemental Figure S7: Meta-analytic average positive predictive value (PPV) of being in the high-risk group (top 0.1% of risk) for legal-involved patients.

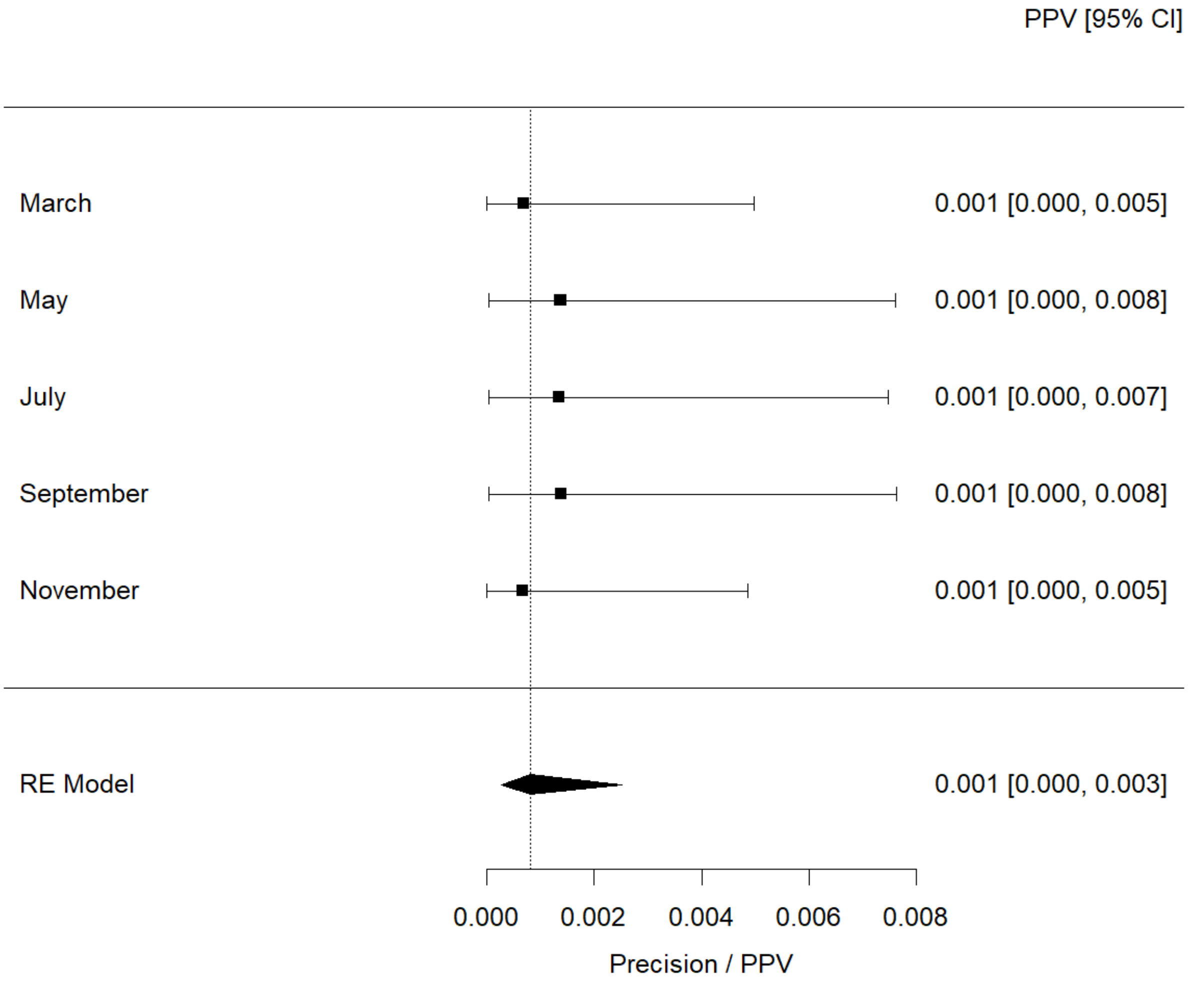

Source/Notes: SOURCE Authors' analysis of data from the VA Corporate Data Warehouse. NOTES In this context, the PPV signifies that 0.1% of legal-involved patients in the high-risk group died by suicide.

Supplemental Figure S8: Meta-analytic average of the false negative rate (FNR) for legal-involved patients.

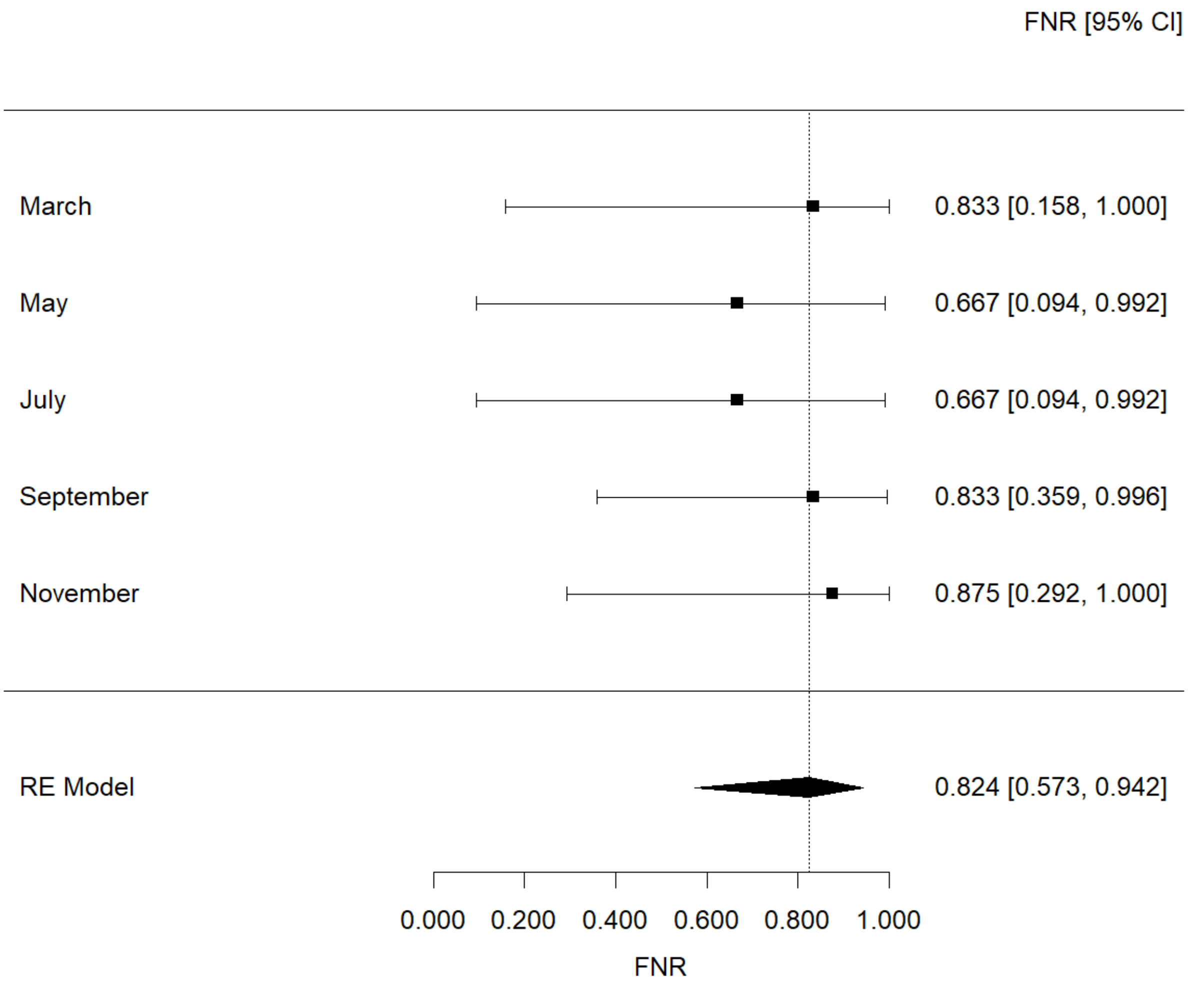

Source/Notes: SOURCE Authors' analysis of data from the VA Corporate Data Warehouse. NOTES In this context, the FNR signifies that 82.4 % of the deaths by suicide among legal-involved patients were in the lower-risk group.

Supplemental Figure S9: Meta-analytic average number of monthly suicide deaths and attempts per 100,000.

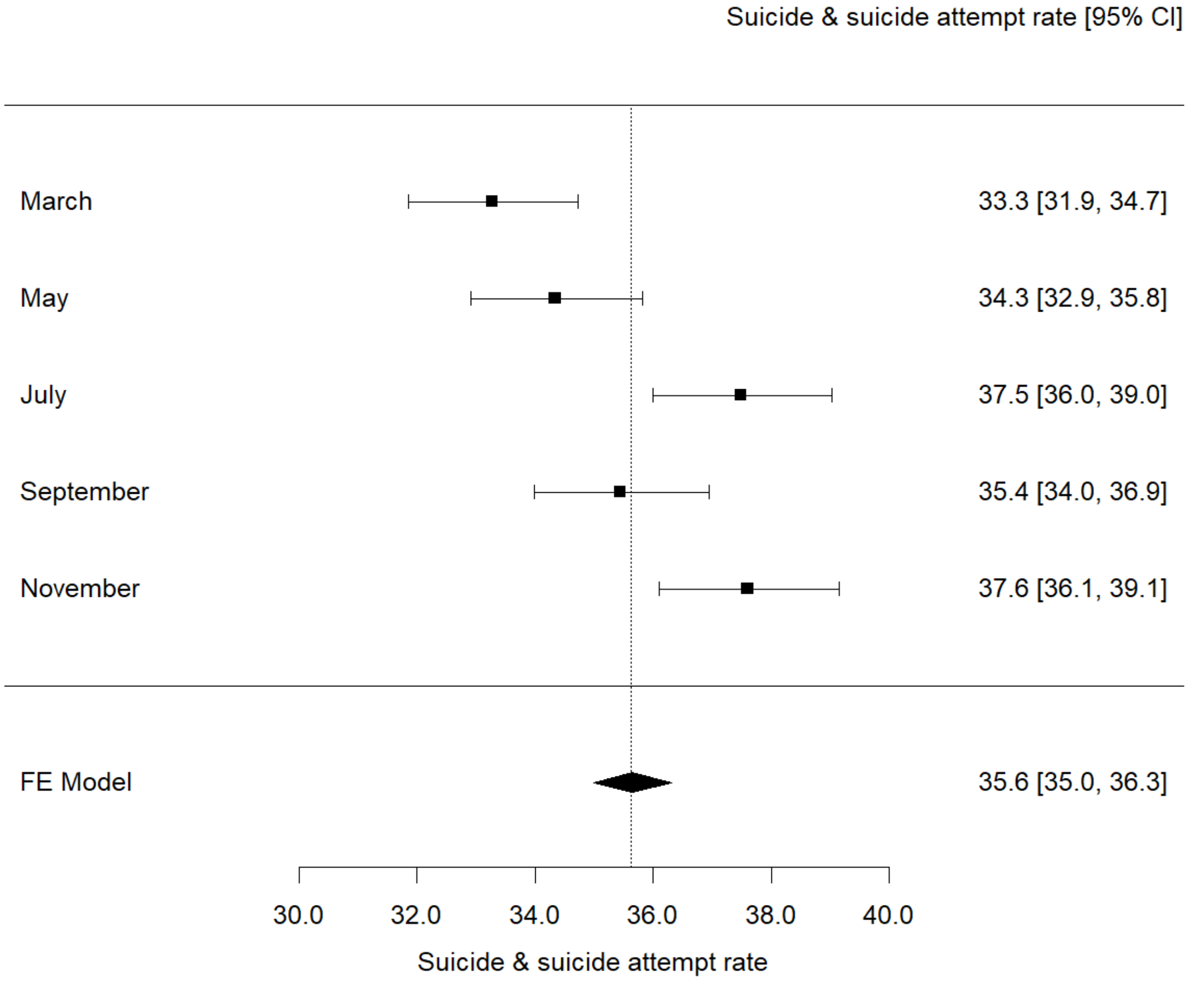

Source/Notes: SOURCE Authors' analysis of data from the VA Corporate Data Warehouse. NOTES The monthly average (35.6) can be multiplied by 12 to get the more common metric of 427.2 annual deaths or attempts per 100,000.

Supplemental Figure S10 Meta-analytic monthly average incident rate ratio ("risk concentration") of high-risk group (top 0.1% of risk) to the rest of the sample.

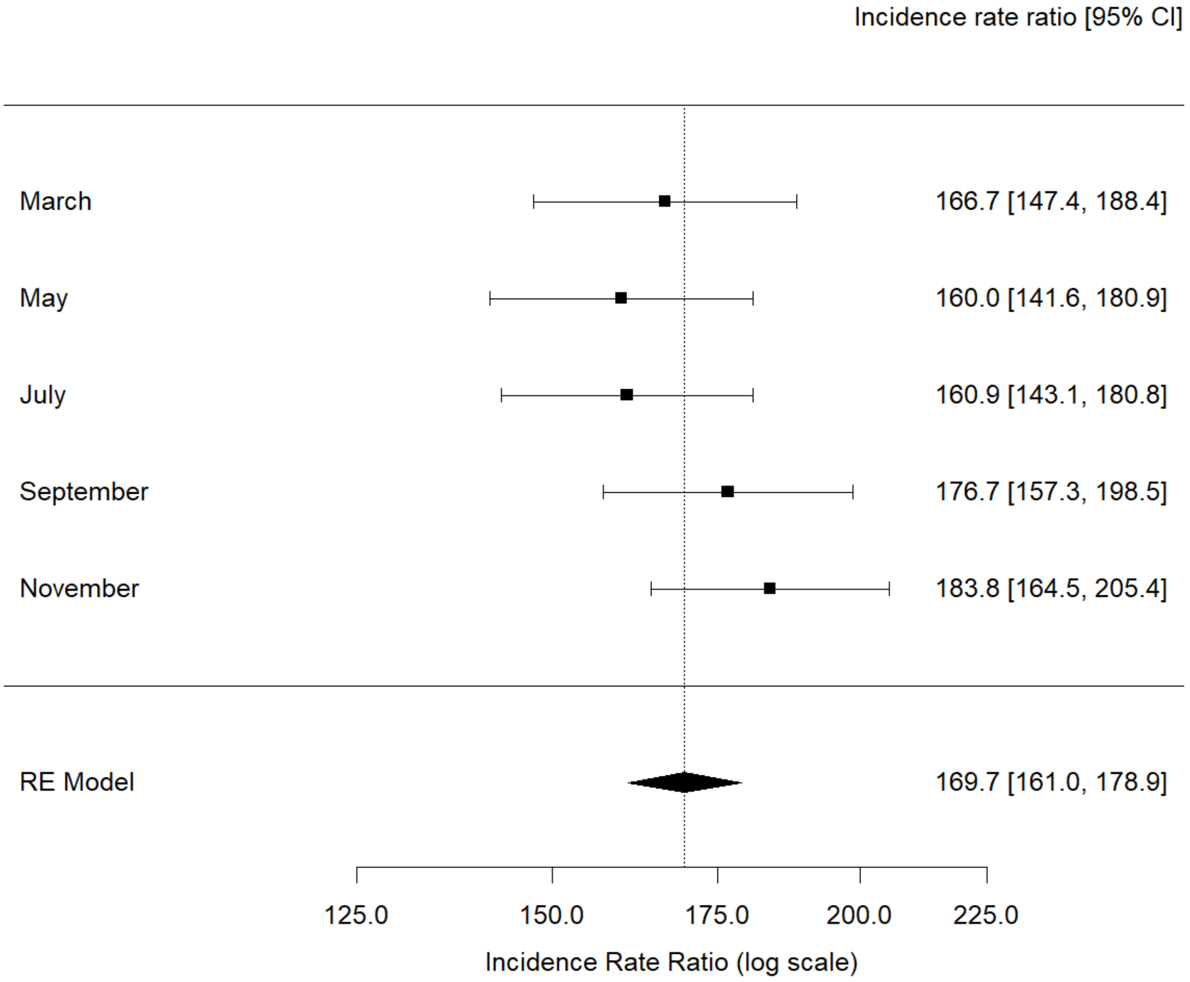

Source/Notes: SOURCE Authors' analysis of data from the VA Corporate Data Warehouse. NOTES In this context, the incident rate ratio signifies that the high-risk group died by or attempted suicide 169.7 times more frequently than the rest of the sample.

Supplemental Figure S11: Meta-analytic average positive predictive value (PPV) of being in the high-risk group (top 0.1% of risk).

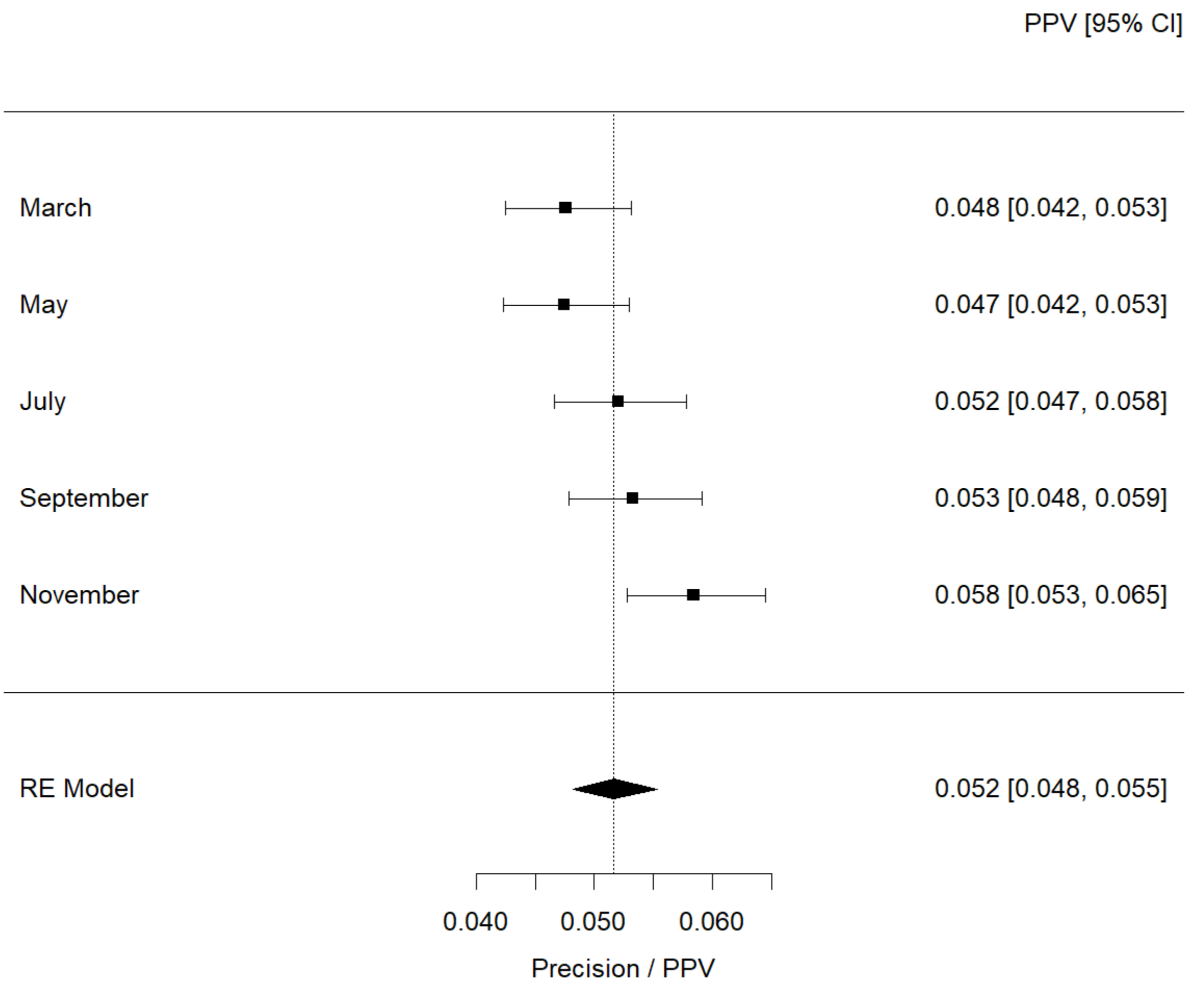

Source/Notes: SOURCE Authors' analysis of data from the VA Corporate Data Warehouse. NOTES In this context, the PPV signifies that 5.2% of those in the high-risk group attempted or died by suicide.

Supplemental Figure S12: Meta-analytic average of the false negative rate (FNR).

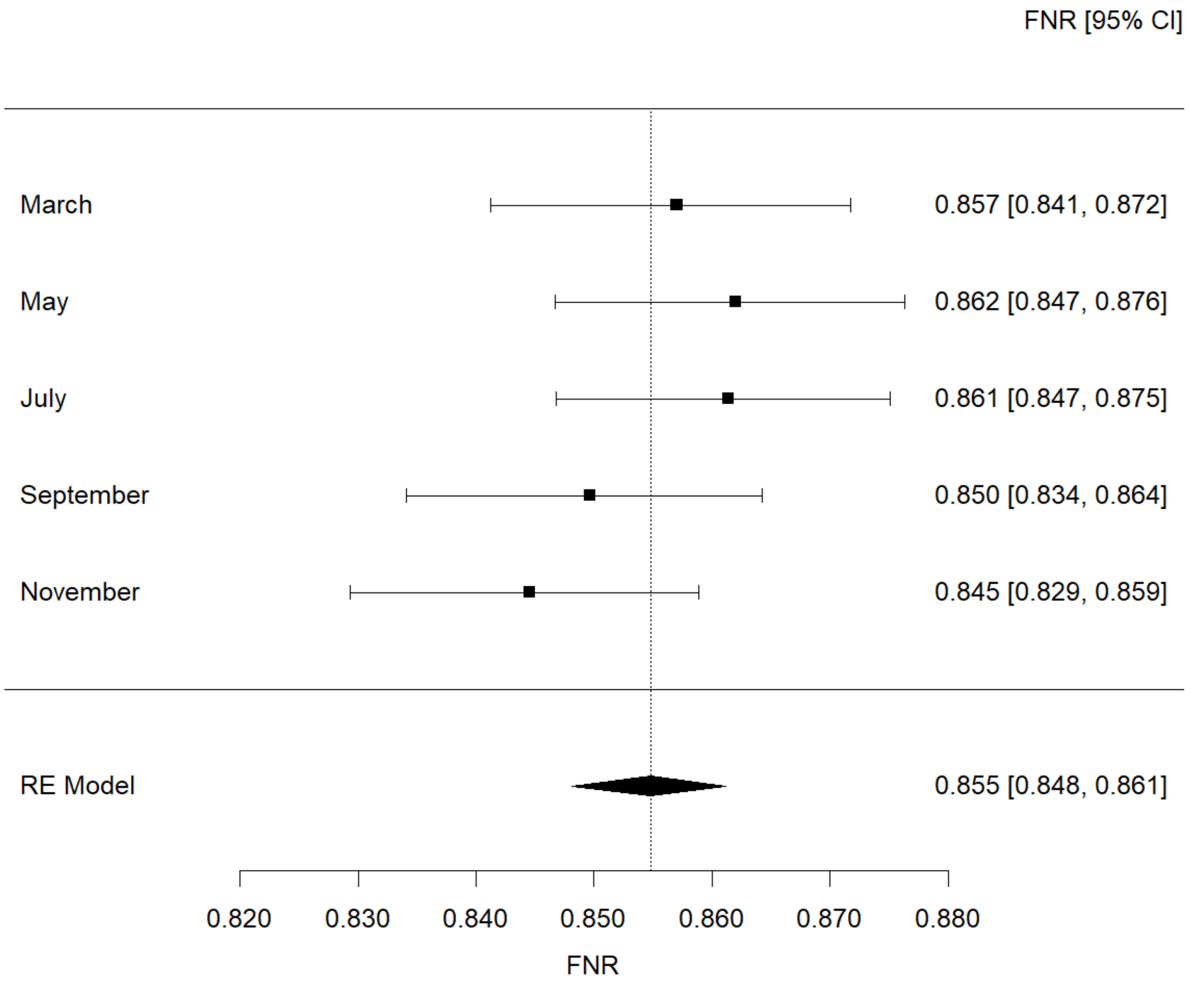

Source/Notes: SOURCE Authors' analysis of data from the VA Corporate Data Warehouse. NOTES In this context, the FNR signifies that 85.5% of the attempts and deaths by suicide were in the lower-risk group.

Supplemental Figure S13: Meta-analytic monthly average incident rate ratio ("risk concentration") of high-risk group (top 0.1% of risk) to the rest of the sample.

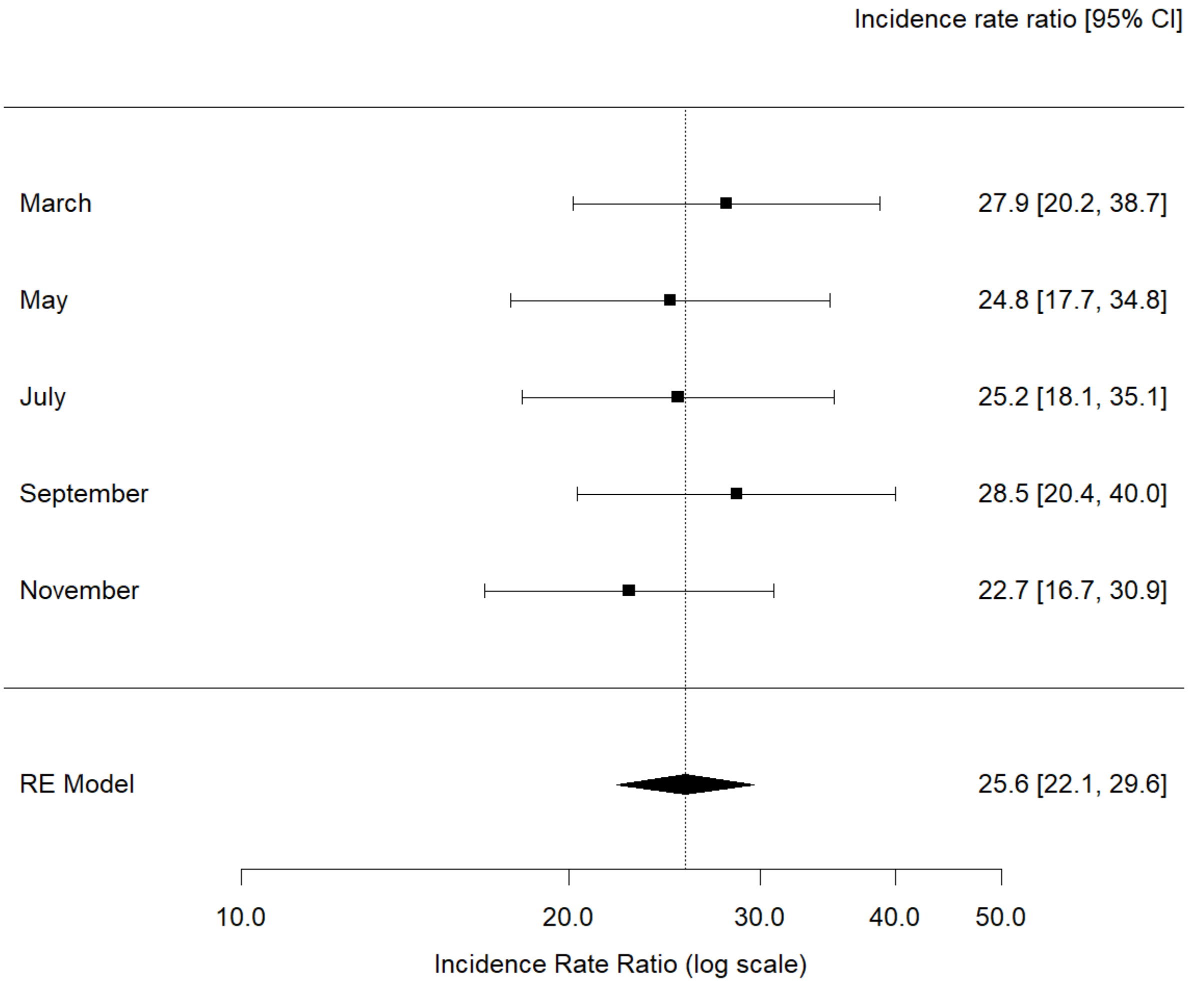

Source/Notes: SOURCE Authors' analysis of data from the VA Corporate Data Warehouse. NOTES In this context, the incident rate ratio signifies that the high-risk group died by or attempted suicide 25.6 times more frequently than the rest of the sample.

Supplemental Figure S14: Meta-analytic average positive predictive value (PPV) of being in the high-risk group (top 0.1% of risk) for legal-involved veterans.

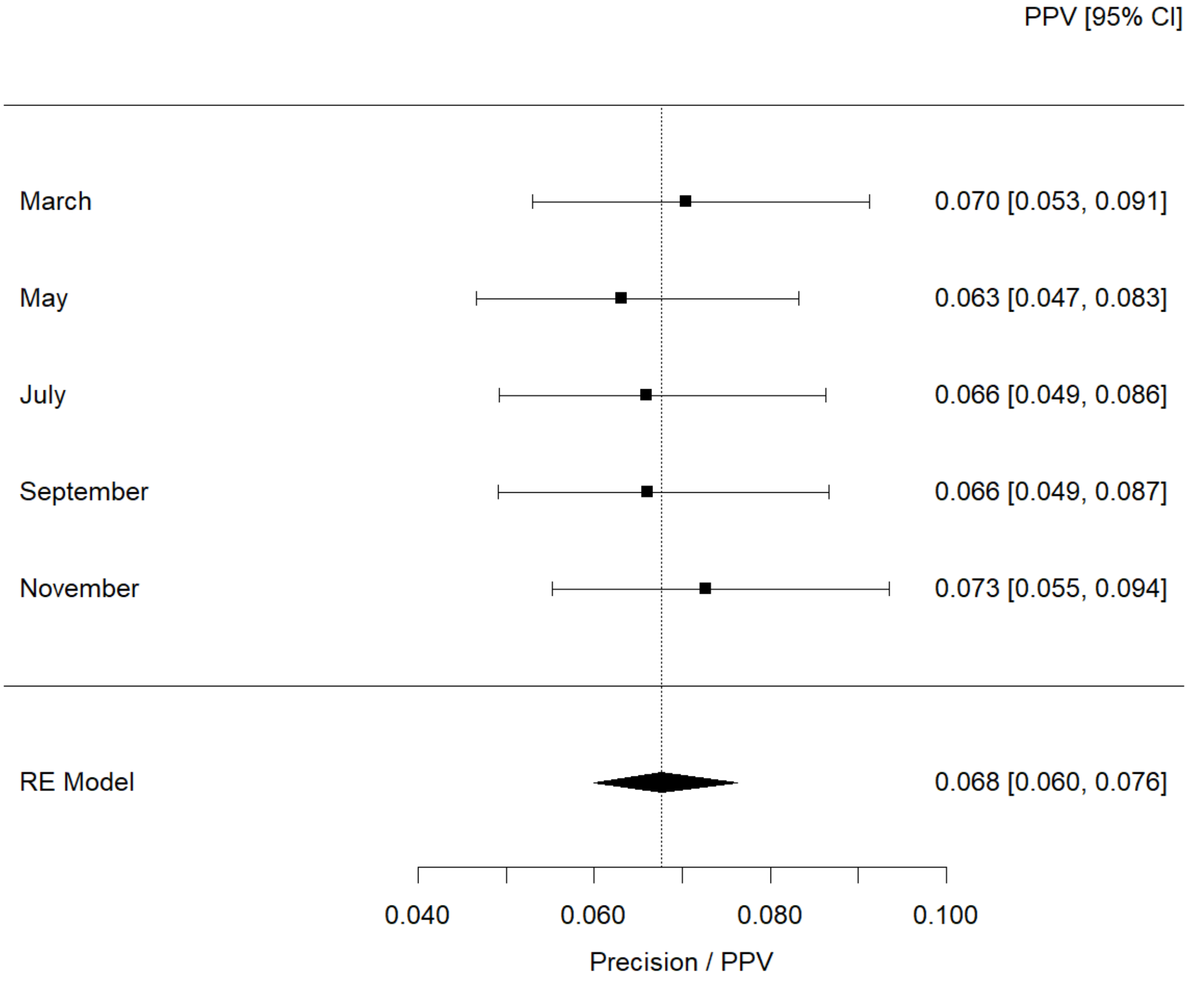

Source/Notes: SOURCE Authors' analysis of data from the VA Corporate Data Warehouse. NOTES In this context, the PPV signifies that 6.8% of those in the high-risk group attempted or died by suicide.

Supplemental Figure S15: Meta-analytic average of the false negative rate (FNR).

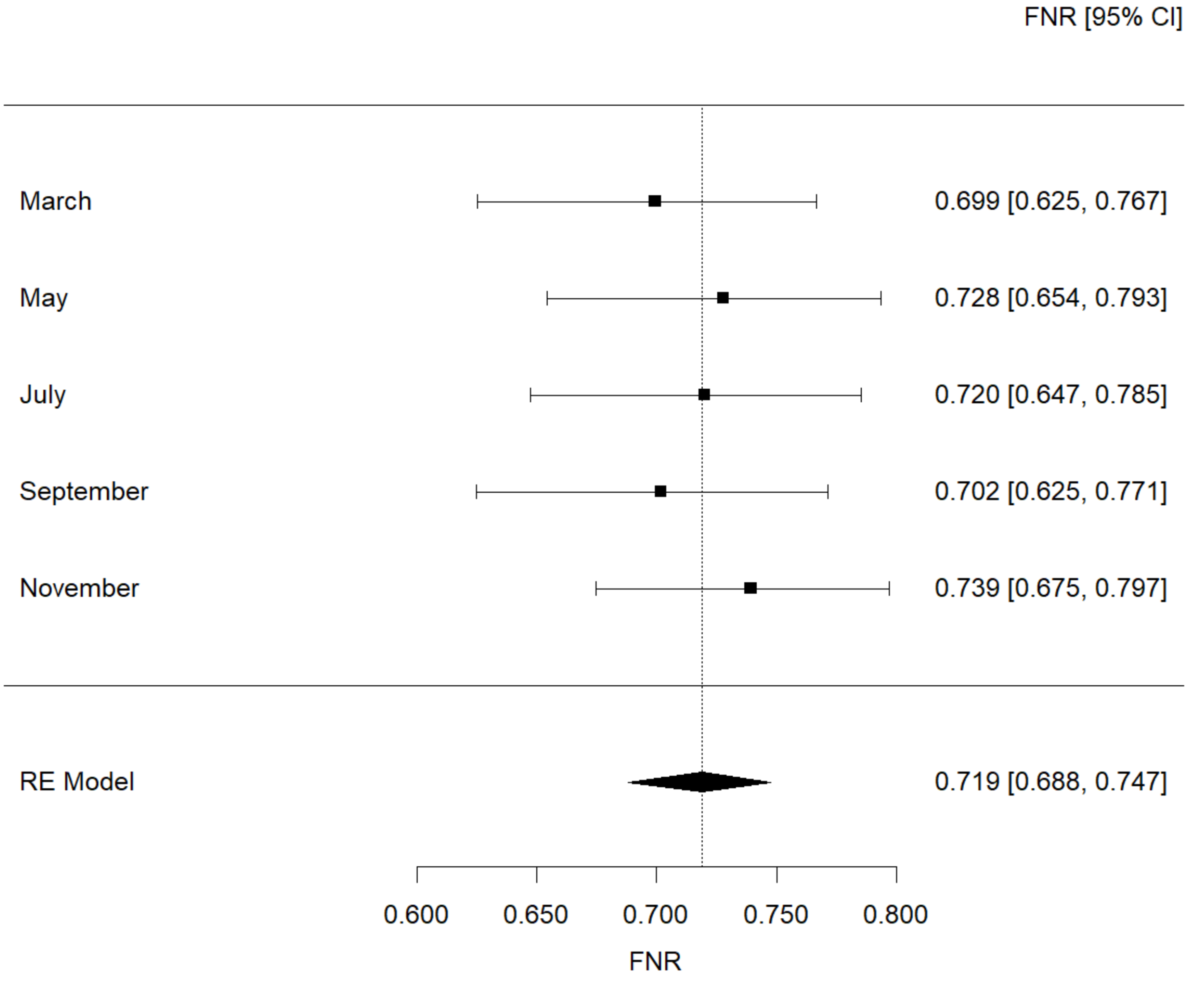

Source/Notes: SOURCE Authors' analysis of data from the VA Corporate Data Warehouse. NOTES In this context, the FNR signifies that 71.9% of the attempts and deaths by suicide were in the lower-risk group.
